# Is aggregated synthetic amorphous silica toxicologically relevant?

**DOI:** 10.1186/s12989-019-0331-3

**Published:** 2020-01-03

**Authors:** Sivakumar Murugadoss, Sybille van den Brule, Frederic Brassinne, Noham Sebaihi, Jorge Mejia, Stéphane Lucas, Jasmine Petry, Lode Godderis, Jan Mast, Dominique Lison, Peter H. Hoet

**Affiliations:** 1 Laboratory of Toxicology, Unit of Environment and Health, Department of Public Health and Primary Care, KU Leuven, 3000 Leuven, Belgium; 2 Louvain centre for Toxicology and Applied Pharmacology, Institute of Experimental and Clinical Research, Université catholique de Louvain, 1200 Brussels, Belgium; 3 Trace Elements and Nanomaterials, Sciensano, 1180 Uccle, Belgium; 4 National Standards, FPS Economy, 1000 Brussels, Belgium; 5 Synthesis Irradiation and Analysis of Materials Platform (SIAM), University of Namur, Rue de Bruxelles 61, 5000 Namur, Belgium; 6 LARN-NARILIS, University of Namur, Rue de Bruxelles 61, 5000 Namur, Belgium; 7 Laboratory for Occupational and Environmental Hygiene, Unit of Environment and Health, Department of Public Health and Primary Care, KU Leuven, 3000 Leuven, Belgium; 8 IDEWE, External Service for Prevention and Protection at work, Interleuvenlaan 58, 3001 Heverlee, Belgium

**Keywords:** Nanomaterials, Synthetic amorphous silica, Aggregates, In vitro toxicity, Biological activity

## Abstract

**Background:**

The regulatory definition(s) of nanomaterials (NMs) frequently uses the term ‘agglomerates and aggregates’ (AA) despite the paucity of evidence that AA are significantly relevant from a nanotoxicological perspective. This knowledge gap greatly affects the safety assessment and regulation of NMs, such as synthetic amorphous silica (SAS). SAS is used in a large panel of industrial applications. They are primarily produced as nano-sized particles (1–100 nm in diameter) and considered safe as they form large aggregates (> 100 nm) during the production process. So far, it is indeed believed that large aggregates represent a weaker hazard compared to their nano counterpart. Thus, we assessed the impact of SAS aggregation on in vitro cytotoxicity/biological activity to address the toxicological relevance of aggregates of different sizes.

**Results:**

We used a precipitated SAS dispersed by different methods, generating 4 ad-hoc suspensions with different aggregate size distributions. Their effect on cell metabolic activity, cell viability, epithelial barrier integrity, total glutathione content and, IL-8 and IL-6 secretion were investigated after 24 h exposure in human bronchial epithelial (HBE), colon epithelial (Caco2) and monocytic cells (THP-1). We observed that the de-aggregated suspension (DE-AGGR), predominantly composed of nano-sized aggregates, induced stronger effects in all the cell lines than the aggregated suspension (AGGR). We then compared DE-AGGR with 2 suspensions fractionated from AGGR: the precipitated fraction (PREC) and the supernatant fraction (SuperN). Very large aggregates in PREC were found to be the least cytotoxic/biologically active compared to other suspensions. SuperN, which contains aggregates larger in size (> 100 nm) than in DE-AGGR but smaller than PREC, exhibited similar activity as DE-AGGR.

**Conclusion:**

Overall, aggregation resulted in reduced toxicological activity of SAS. However, when comparing aggregates of different sizes, it appeared that aggregates > 100 nm were not necessarily less cytotoxic than their nano-sized counterparts. This study suggests that aggregates of SAS are toxicologically relevant for the definition of NMs.

## Background

Synthetic amorphous silica (SAS) represents a group of nanomaterials (NMs) manufactured either by thermal (pyrogenic/fumed) or wet route (precipitated, gel and colloidal) [1]. SAS is primarily produced as nano-sized primary particles (1–100 nm in diameter) that form micron-sized aggregates and agglomerates of aggregates during their production process [2, 3]. Aggregates are composed of particles that are strongly linked by chemical bonding, whereas, in agglomerates, the particles/aggregates are bound together by reversible weak forces such as charge and Van der Waals interactions [4]. Thus aggregates are the smallest secondary structure of SAS in their manufactured form.

SAS is widely used in industrial applications ranging from food (food additive E551) and cosmetics to pharmaceutical applications [1, 5–8]. SAS is traditionally considered as non-toxic due to their amorphous nature and several authors argued that it is very unlikely they pose health hazards as they are aggregated in structures of larger sizes [3, 9, 10]. However, our recent reviews indicate that all types of SAS cause acute effects such as cytotoxicity in several cell types mainly via the induction of oxidative stress and/or pro-inflammatory responses [1, 11]. Some forms of SAS, such as colloidal SAS, have also the potential to induce DNA damage in cell cultures. However, while these studies focus on the hazard of nano-sized SAS, influence of SAS aggregation on cytotoxicity/biological acitivity remains unexplored.

In the recommended regulatory definition of NMs by the European Union (EU), an important aspect is the use of the term “aggregates”. It states that “*manufactured material containing particles, in an unbound state or as an aggregate or as an agglomerate and where, for 50 % or more of the particles in the number size distribution, one or more external dimensions is in the size range 1 nm-100 nm*” [12]. There is paucity of evidence that AA are significantly relevant from a nanotoxicological perspective.

Here we aim to determine the impact of SAS aggregation on their cytotoxicity/biological activities, and to determine the toxicological relevance of aggregates of different sizes using in vitro cell cultures. The most quoted nanotoxicity paradigm is “the smaller the size of the nanoparticles the greater the toxicity/biological responses” [13–16]. *Therefore*, *we hypothesized that, small aggregates of SAS induce stronger cytotoxicity compared to their larger counterparts.* We used a precipitated amorphous silica (primary particle size 14–23 nm), which is a representative of SAS used as food additives [3]. Using different dispersion methods, we generated four suspensions with different aggregate size distributions and tested their cytotoxicity/biological activities in several cell types. Dispersions included de-aggregated (by sonication) and aggregated (vortexed) SAS, as well as fractionation of the aggregated suspension to separate rapidly precipitating aggregates from others that remain in suspension in the dispersion medium.

## Results

### Dispersion of SAS

**Fig. 1:**
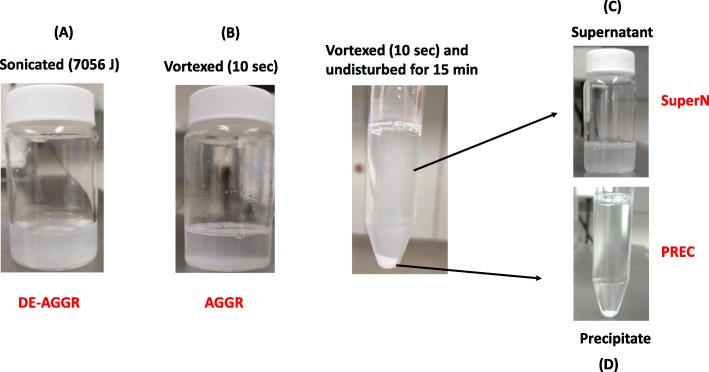
Preparation of synthetic amorphous silica suspensions (SAS). DE-AGGR - 2.56 mg/mL of SAS sonicated with an energy of 7056 J (**a**); AGGR - 2.56 mg/mL of SAS vortexed for 10 s (**b**); SuperN (0.64 mg/mL) - AGGR dispersion left undisturbed for 15 min after vortexing and fractionated in supernatant (**c**) and PREC (1.92 mg/mL) - precipitates of AGGR (**d**)

Figure 1 shows how the four different suspensions of SAS were freshly prepared before each independent experiment. The ‘DE-AGGR’ suspension was prepared using the generic NANOGENOTOX protocol [17] delivering the energy of 7056 J to the suspension by sonication. The ‘AGGR’ suspension was prepared in the same dispersion medium and vortexed for 10 s. The supernatant ‘SuperN’ and precipitate ‘PREC’ suspensions were obtained from the ‘AGGR’ suspension by leaving it undisturbed for 15 min (see materials and methods).

### Size characterization of SAS suspensions

**Fig. 2:**
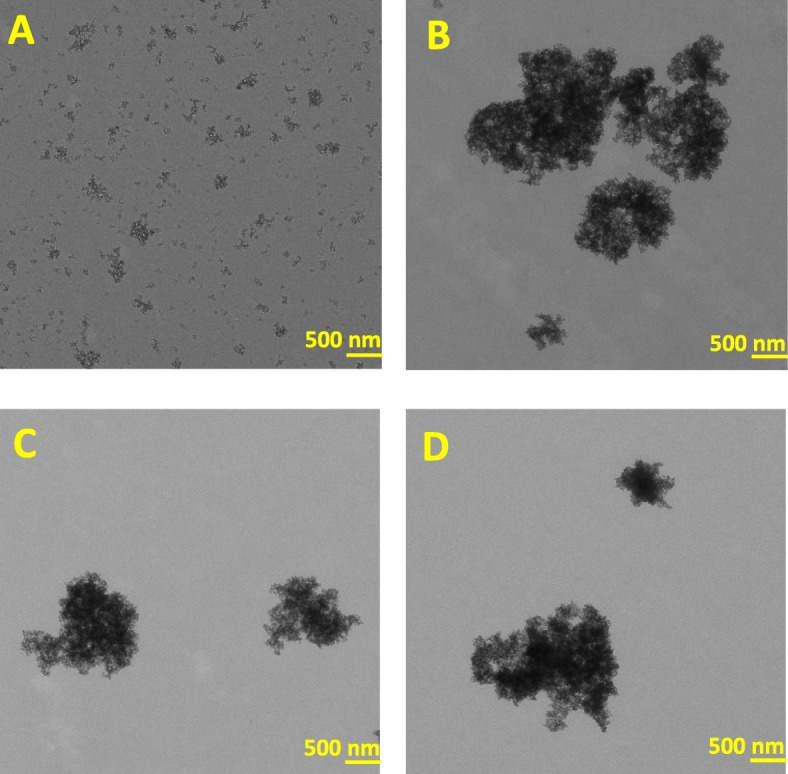
Representative TEM micrographs of freshly prepared SAS stock suspensions. DE-AGGR (**a**), AGGR (**b**), SuperN (**c**) and PREC (**d**)

Transmission electron microscopy (TEM) micrographs revealed that SAS in the DE-AGGR fraction (Fig. 2a) were much less aggregated than in AGGR (Fig. 2b), SuperN (Fig. 2c) and PREC (Fig. 2d). Aggregates in all suspensions showed a fractal-like structure. Table 1 shows the size of SAS in the different suspensions measured by TEM and dynamic light scattering (DLS). The mean equivalent circle diameter (ECD) was 100 nm for DE-AGGR and 2000 nm for AGGR. The mean hydrodynamic diameter (Z-average) of DE-AGGR was 264 nm and 10 μm for AGGR. Feret min, which describes the smallest external dimension according to the EU definition, was 28 nm and 600 nm for DE-AGGR and AGGR SAS, respectively. Feret min and ECD analysis was not possible for PREC and SuperN due to their low stock concentration, therefore, the sizes were manually measured (arbitrary line measurement) and determined as 600 and 750 nm, respectively; Z-average of SuperN and PREC suspension was around 3953 nm and 3332 nm. Measurements by centrifugal liquid sedimentation (CLS), given its larger useful range, is somewhat less dependent on the stability of the samples. Therefore, CLS was used to measure the approximate sizes of SuperN and PREC aggregates and the results revealed some slight differences between SuperN and PREC. SuperN was mainly composed of one size population (mean size 4580 nm) and PREC of two populations (3900 nm and 6370 nm) (data not shown in the table). In conclusion, a clear difference in size distribution was observed for DE-AGGR and AGGR. Although SuperN and PREC appeared visually different (Fig. 1), such difference could not be established using TEM, DLS or CLS.

**Table 1:** Size characterization of SAS dispersed in 0*.05% Bovine Serum Albumin (BSA)*

Stock suspensions	Stock concentration (mg/mL)	TEM		DLS
		Mean ECD (nm)	Mean Feret min (nm)	Z-average (nm)
DE-AGGR	2.56	100 ± 14	28 ± 0.6	264
AGGR	2.56	2000	600	12,530
SuperN	0.64	600^a^	n/a	3953
PREC	1.92	750^a^	n/a	3332

Mean equivalent circle diameter (ECD) and mean feret minimum (Feret min) measured by TEM; Mean hydrodynamic diameter (Z-average) by DLS; n/a-not available. ^a^size was measured manually using the arbitrary line measurement tool of the iTEM software

**Fig. 3:**
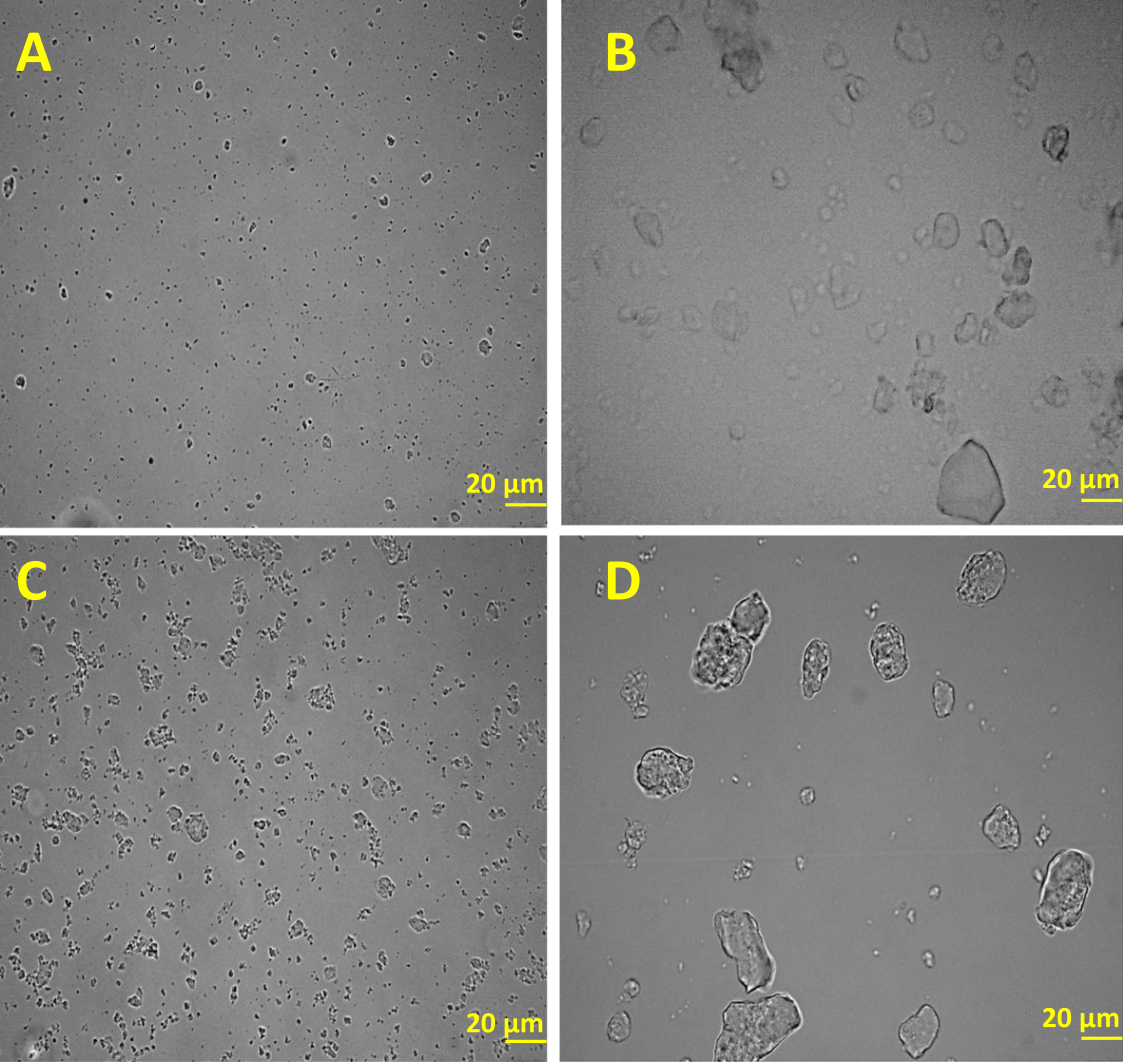
Representative bright field microscopic images of freshly prepared SAS stock suspensions. DE-AGGR (**a**), AGGR (**b**), SuperN (**c**) and PREC (**d**). Scale bar -20 μm

To establish the difference between PREC and SuperN, images were taken using bright field microscopy (Fig. 3). Pictures indicate that the aggregates in PREC (Fig. 3d) are much larger than the aggregates in SuperN (Fig. 3c) and DE-AGGR (Fig. 3a). Using an arbitrary line measurement tool, the size of 100 aggregates from representative pictures were measured manually. The mean size of PREC aggregates was roughly about 25 μm in diameter while SuperN aggregates measured only 2.5 μm. In conclusion, we showed by different approaches that all suspensions generated in this study were composed of aggregates of different size distributions.

The stability of the suspensions in exposure media was measured by DLS (Table 2). The stock suspensions were diluted in different exposure media and mean sizes were measured directly and after 24 h. Z-average of DE-AGGR SAS in DMEM/F12 (used for HBE cell cultures) and RPMI 1640 (used for THP-1) exposure media was approximately 225 nm and remained unchanged after 24 h except in DMEM/HG medium (used for Caco2), in which Z-average increased to 1275 nm. The mean size of SuperN was 4 μm at the beginning, and did not drastically change after 24 h (except in DMEM/HG). Although the measurements of AGGR and PREC do not reflect the size of aggregates, the results are given in the table for completeness.

**Table 2:** Size characterization of SAS in stock suspensions and exposure media (for H*BE, Caco2 and THP-1) using DLS*

		Stock		HBE		Caco2		THP1	
AM		Z-avg	PDI	Z-avg	PDI	Z-avg	PDI	Z-avg	PDI
DE-AGGR	0 h	264	0.33	228	0.35	225	0.34	228	0.36
	24 h	260	0.32	233	0.37	1275	0.28	226	0.42
AGGR	0 h	10,040	0.90	3212	0.72	4321	0.82	3566	0.97
	24 h	12,374	0.88	3059	0.80	1637	0.46	2688	0.75
SuperN	0 h	3953	0.94	4507	0.87	4500	0.89	4507	0.95
	24 h	3661	1.00	3851	0.83	2233	0.85	3866	0.95
PREC	0 h	3332	0.90	1101	0.85	1370	0.83	1561	0.93
	24 h	4329	0.96	957	0.88	1376	0.82	955	0.91

Freshly prepared stock suspensions were diluted to 100 μg/mL in different exposure media (without serum) and, hydrodynamic sizes (Z-avg) and polydispersity index (PDI) were measured directly and after 24 h (incubated at 37 °C)

### Si quantification in SAS SuperN and PREC suspensions

The quantification of Si by inductively coupled plasma mass spectrometry (ICP-MS) in SuperN and PREC suspensions derived from the AGGR suspension indicated that Si was distributed in each fraction at a ratio of 24.7% ± 0.67 (mean ± SEM) in SuperN and 75.3% ± 0.67 for PREC. Therefore, the mass concentration of PREC and SuperN suspensions were determined as 1.92 mg/mL and 0.64 mg/mL, respectively.

### Influence of aggregation on in vitro dosimetry

**Fig. 4:**
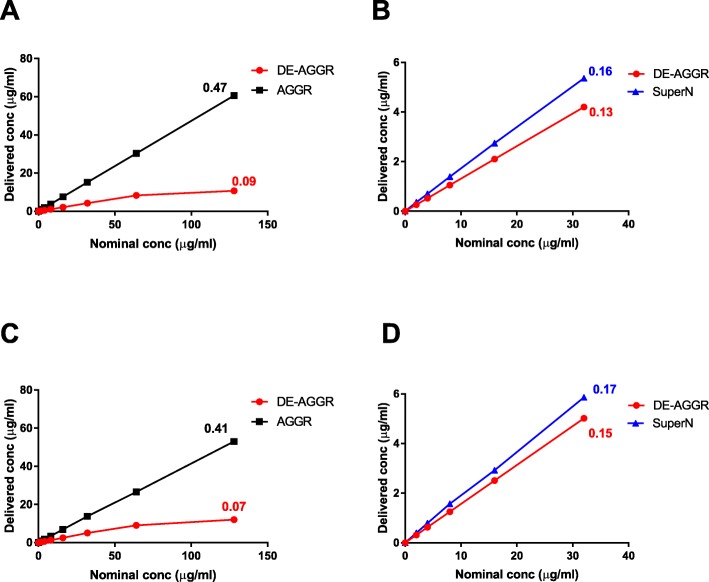
Estimated concentration reaching the bottom of the wells after 24 h as a function of nominal concentrations applied in cell based assays. Dosimetry simulation was performed by a distorted grid (DG) model and compared for different SAS suspensions using parameters obtained from exposure media DMEM/F12 (**a** and **b**) and RPMI 1640 (**c** and **d**). The slope values are indicated near the respective lines. *R*^2^ > 0.9 for all the suspensions. The percentage of dose delivered to the cells did not differ in 96 or 24 well plates, as the height of the liquid column was equal (6 mm)

In vitro dosimetry simulation was performed using DMEM/F12 (used for HBE cell cultures) and RPMI 1640 (THP-1) because SAS diluted in DMEM/HG (Caco2) exposure medium agglomerated further over 24 h incubation (change in agglomerate size > 30%) and did not meet the criteria to apply the volume centrifugation method (VCM) method. Additional file 1: Table S1 lists the main parameters used to perform the dosimetry simulation and Fig. 4 shows the estimated SAS concentration reaching the bottom of the wells as a function of nominal (applied) dose. Regardless of the exposure media, nearly 7–9% of the DE-AGGR applied concentration reached the cells while it was at least 5-fold higher for AGGR SAS (41–47%) at the simulated concentrations (Fig. 4a and c). It is important to mention here that the delivered concentrations for AGGR could be underestimated since the DLS size used for dosimetry simulation did not reflect the PREC aggregates in AGGR suspension. Based on ICP-MS analysis, we found that the highest concentration tested for SuperN suspension in biological experiments was 32 μg/mL. Therefore, the dosimetry for SuperN was simulated for nominal concentrations from 2 to 32 μg/mL and compared with DE-AGGR (Fig. 4b and d). The delivered dose between these suspensions was not substantially different. The percentage of concentrations delivered to the cells also did not differ much for 96 and 24 well plates as the height of the cell culture medium was similar (6 mm).

### Comparison of in vitro biological responses

Food additive SAS are produced in large quantity. Therefore, in addition to ingestion, exposure via inhalation during production and processing is also inevitable. Therefore, we used both a human bronchial (16HBE14o- or HBE) and colon epithelial cell line (Caco2) to investigate the cytotoxic effects in vitro. In addition, we used a human monocytic cell line (THP-1) as monocytes/macrophages are the first line of defence once particles enter into the body. Physiologically relevant doses for short term exposure (0–128 μg/mL) estimated from Occupational Exposure Limits (OELs) of amorphous silica were used in this study [11]. To investigate the short-term cytotoxicity/biological activity in vitro, we assessed different endpoints including cytotoxicity, oxidative stress, epithelial barrier integrity and pro-inflammatory responses.

Since this study aims to compare the magnitude of responses induced by aggregates of different sizes, we first selected endpoints for which a significant response was recorded (Table 3). If no impact of SAS treatment on a given endpoint was measured, this endpoint was not further investigated. In a second step, we analysed only the endpoints showing a significant difference after exposure to SAS. Detailed data for all the endpoints are presented only in Additional file 1: Figure S1-S3.

**Table 3:** Summary of the in vitro responses to SAS exposure

Biological endpoints	AGGR			DE-AGGR			SuperN			PREC		
	HBE	Caco2	THP1	HBE	Caco2	THP1	HBE	Caco2	THP1	HBE	Caco2	THP1
Cell metabolic activity	No	Yes	Yes	Yes	Yes	Yes	No	Yes	Yes	No	No	Yes
Cell viability	Yes	No	Yes	Yes	Yes	Yes	No	Yes	Yes	No	No	No
GSH	Yes	Yes	Yes	Yes	Yes	Yes	Yes	Yes	Yes	No	No	No
TEER	Yes	No	n/a	Yes	Yes	n/a	Yes	Yes	n/a	No	No	n/a
IL-8	No	Yes	Yes	Yes	Yes	Yes	Yes	Yes	Yes	Yes	No	No
IL-6	No	No	No	Yes	Yes	No	Yes	No	No	Yes	No	No

“Yes” indicates *p* < 0.05 (One-way ANOVA) and there is a significant difference compared to control at any of the tested concentrations; “No” indicates when *p* > 0.05 and no difference compared to control; n/a - not available

To determine the impact of aggregation, we first compared DE-AGGR and AGGR suspensions using two-way ANOVA. If differences were observed, a post hoc test (Bonferroni’s multiple comparison test) was used to determine which suspension induced a stronger effect at the same mass concentrations (Table 4a). When comparing DE-AGGR vs AGGR, there was a significant difference in 14 comparisons out of 16, with DE-AGGR inducing more pronounced effects than AGGR (see Table 4a and Additional file 1: Figure S1).

**Table 4:**
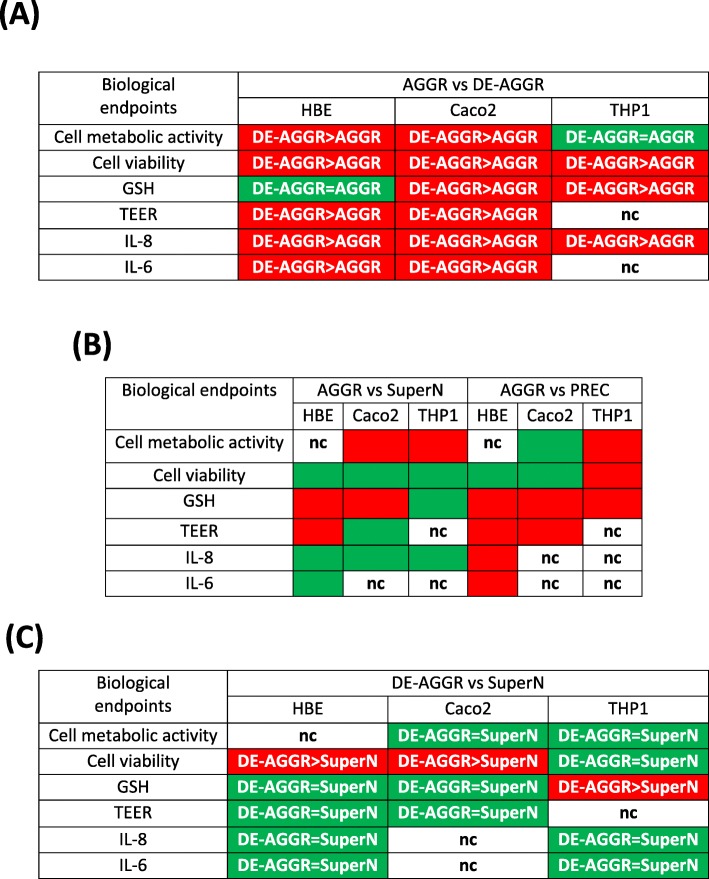
Summary of in vitro responses to differently aggregated SAS suspensions

“Green” indicates *p*>0.05 (Two-way ANOVA) and no significant difference between the suspensions; “Red” indicates *p*<0.05 and a significant difference between the suspensions - in this case, Bonferroni’s multiple comparison test was used to statistically determine which suspension induced the strongest effect; nc-not compared as both suspensions did not induce any significant activity compared to control

Secondly, we compared AGGR with SuperN and PREC (see Table 4b and Additional file 1: Figure S2). It is important to mention here that the concentrations of SuperN and PREC suspensions were not adjusted to the AGGR concentrations in this analysis (please refer to the in vitro exposure conditions for explanation), which allows to determine which of the SuperN or PREC suspension accounts more for the effects induced by AGGR. When comparing AGGR vs SuperN, 9 out of 14 comparisons indicate that AGGR suspensions induced similar effects as SuperN (no significant difference). When comparing AGGR vs PREC, 9 out of 12 comparisons showed that AGGR induced significantly stronger effects compared to PREC. These results suggest that the SuperN fraction accounts more for the effects induced by AGGR than PREC.

Finally, to determine the toxic potency of SuperN aggregates in comparison to the nano-sized aggregates, we compared SuperN (the dose was adjusted based of ICP-MS measurements) with DE-AGGR at mass concentrations between 2 and 32 μg/mL. The summary of the results are presented in Table 4c and Additional file 1: Figure S3. Intriguingly, most comparisons (11 out of 14) indicated that SuperN and DE-AGGR induced similar effects except for the LDH assay in HBE and Caco2, and GSH depletion in THP-1, where DE-AGGR was more potent than SuperN.

## Discussion

To determine the impact of NM aggregation, we compared the in vitro cytotoxicity/biological activities induced by de-aggregated SAS (DE-AGGR) produced by sonication at 7056 J and aggregated SAS (AGGR) in a vortexed suspension. The AGGR SAS was also fractionated into two suspensions based on aggregate sedimentation (PREC and SuperN) and we also, compared their cytotoxicity/biological activities separately and compared to AGGR and DE-AGGR. Overall, aggregation resulted in reduced toxicological activity of SAS. We observed that large precipitating aggregates (PREC) were the least cytotoxic/biologically active. Interestingly, aggregates in SuperN, which were larger (> 100 nm) than DE-AGGR aggregates (< 100 nm), exhibited similar activity as DE-AGGR.

Preparation of the suspensions with aggregates of different sizes were crucial in our study, but no standardized protocol with minimal changes in dispersion medium has been developed so far [1, 11]. Therefore, we explored the potential of applying different levels of energy by sonication to obtain different aggregate sizes. This indeed resulted in de-aggregation of SAS but did not yield sizes with substantial differences whatever the energy delivered (≥ 882 J, see Additional file 2: Figure S4). A recent study also showed that different sonication energies used to disperse SAS did not strongly affect the size distribution and hence the biological effects [18]. Therefore, we used a mechanically vortexed suspension (AGGR) to obtain a size distribution different from DE-AGGR. European Food Safety Authority (EFSA), in its recent scientific opinion, insisted that the size distribution used so far in toxicological studies (mostly de-aggregated NPs like the DE-AGGR suspension presented here), might not be fully representative of SAS in their pristine form [19]. Therefore, the AGGR suspension produced by vortexing in this study might represent a better model of SAS in their pristine (manufactured) form.

The size characterization of SAS suspensions was not straightforward and technically challenging due to the polydisperse size distribution. The PREC fraction of AGGR, predominantly composed of large aggregates (few tens micrometers), did not appear different from SuperN fraction based on TEM, DLS or CLS. This can due to the fact that the PREC aggregates are not dispersed properly for TEM or DLS measurements. For TEM grid preparations, grid-on-drop method was used (refer to [20] for method description) and possibly, this method did not allow very large aggregates to attach to the EM grid due to their quick sedimentation/precipitation. For DLS, very large aggregates might also have sedimented very rapidly and were missed in the analysis. Therefore, measurements for AGGR and PREC might actually not reflect reality. The CLS technique, which separate particles/aggregates by sizes using centrifugal sedimentation, also did not detect these very large aggregates at the lowest centrifugal speed possible because of its limitation to detect particles/aggregates > 20 μm. However, aggregates > 20 μm were easily detected using bright field microscopy. In conclusion, it appeared that a panel of techniques are required to reveal the true size distribution of polydispersed NMs containing large aggregates.

SAS in their pristine form, exist as aggregates of few hundred nanometers to few hundred micrometers [2, 3]. Size characterization of AGGR suspension indicated that the particles were not only strongly aggregated but also showed a high polydispersity in size. On the other hand, DE-AGGR SAS were prepared by sonication, which is the standard technique to prepare well-dispersed (least aggregated or agglomerated) suspensions and to characterize the hazard of nanoparticles. Size distribution analysis of DE-AGGR showed that particles in this suspension were in their least aggregated form compared to other suspensions, which is consistent with the results obtained using the same dispersion protocol [21]. In addition, though dosimetry simulation indicated that much higher dose was delivered to cells for AGGR compared to DE-AGGR SAS, AGGR SAS were consistently less active in cell based assays than DE-AGGR. Furthermore, although changes in SAS behaviour (further agglomeration) was noticed when different biological exposure media were used, the activity was not much influenced by the cell types. This indicates that the changes in particle characteristics in exposure media and delivered doses were not essential confounders for differential responses observed with these suspensions.

While preparing AGGR suspensions, we noticed two fractions, one that quickly sedimented in a few min (PREC) and the other that remained in suspension (SuperN) for hours. Hence we studied their activity separately. Large aggregates in the PREC suspension did not show any or a weak biological activity although its mass concentration was 75% of the AGGR suspension (Table 3). Rapidly sedimenting agglomerates were reported to be more cytotoxic than the one that sediment slowly [22, 23]. However, in the present study, rapid sedimentation and hence high local concentration of large aggregates, did not result in cellular damage, possibly because these aggregates were too large for cellular interaction and uptake, and/or might not demonstrate any nanoscale associated properties.

SuperN aggregates, which were larger than DE-AGGR, showed similar cytotoxic/biological activity as DE-AGGR. Most importantly, delivered doses was not a confounder when comparing AGGR and SuperN aggregates. These findings are somewhat in contrast with studies demonstrating that sub-micron and micron sized silica particles were less potent compared to nano-sized silica [13, 24, 25], suggesting that the size alone may not be necessarily the key determinant of toxicity of aggregated SAS. Apart from size, other physico-chemical characteristics of nanoparticles such as shape also influence the cellular interactions and toxicity. Particles with sharper angular features or elongated were efficiently taken up by cells compared to the spherical ones [26–28]. In this study, SuperN aggregates were mostly non-spherical and exhibited fractal-like structure. Further, despite the fact that the size of these aggregates is far greater than the nano-range, they may still possess the surface of the primary particles. Thus, physico-chemical characteristics such as shape, surface reactivity and stability in aqueous suspension, might contribute to the toxic potential.

The physicochemical properties of different types of silica NM might be different [1] and hence their biological activity [29]. Therefore, the results of this study may only be applicable to the precipitated silica used here, although this approach can be applied to investigate the toxicity of other types of SAS or other NMs.

## Conclusion

This study aimed to assess the impact of aggregation on cytotoxic/biological activities in submerged cell cultures and the toxicological relevance of SAS aggregates of different sizes. In general, aggregation resulted in reduced toxicological activity of SAS. Looking closer, the large, quickly precipitating aggregates (PREC) exhibited the lowest cytotoxicity/biological activity in vitro. On the other hand, an important fraction (25% of the mass of AGGR) of non-precipitating aggregates (SuperN), which contains aggregates larger than 100 nm, exhibited similar activities as nano-sized aggregates (DE-AGGR). We conclude that aggregates with size greater than 100 nm should not be necessarily considered as less toxic than their nano-sized counterparts. This study suggests that aggregates of SAS are toxicologically relevant and should be part of the definition of NMs.

## Materials and methods

### SAS NMs

The European Commission’s Joint Research Centre (JRC, Italy) kindly provided the representative NM silica JRCNM02000. Detailed physico-chemical characterization in their manufactured form is provided in the technical report of JRC [21].

### Dispersion of SAS

The ‘DE-AGGR’ suspension was prepared using the generic NANOGENOTOX protocol [17]: 15.36 mg SAS were pre-wetted with 30 μL EtOH and then suspended in 5.970 mL bovine serum albumin (BSA) 0.05% (2.56 mg SAS/mL, final volume 6 mL). Then the suspension was sonicated for 16 min (energy 7056 joules, Microson XL 2000, 3 mm probe, Belgium).

The ‘AGGR’ suspension was prepared as follows: 15.36 mg SAS were pre-wetted with 30 μL EtOH and then suspended in 5.970 mL BSA 0.05% (2.56 mg SAS/mL, final volume 6 mL). This suspension was then vortexed for 10 s.

The ‘SuperN’ and ‘PREC’ suspensions were obtained from the ‘AGGR’ suspension by leaving it undisturbed for 15 min: the supernatant (5.8 mL) was removed and 200 μL of 0.05% BSA was added (‘SuperN’ fraction, final volume 6 mL); and 5.8 mL 0.05% BSA was added to the precipitate (‘PREC’ fraction, final volume 6 mL), which was redispersed by vortexing for 10 s.

### Dynamic light scattering (DLS)

DLS measurements were performed using a ZetaSizer Nano ZS instrument (Malvern Instruments, Malvern, UK) to evaluate the size distribution of SAS in suspensions. Freshly prepared stock suspensions and SAS in cell exposure medium (100 μg/mL) were tested for each condition. The settings were 1.544 for the refractive index and 0.2 for the absorption parameter. The selected dispersant was water (refractive index, 1.33). The mean hydrodynamic diameter (Z-average) and the polydispersity index (PDI) were measured using the version 7.11 of the Zetasizer software.

### Transmission Electron microscopy (TEM)

TEM specimens of freshly prepared suspensions were prepared by the “grid on top”method and examined using a well-aligned Tecnai Spirit microscope (FEI, Eindhoven, Netherlands) operating at 120 kV, at a spot size 3 and imaged in BF-mode in parallel beam conditions. Images were typically recorded at approximately 500 nm below minimal contrast conditions. Digital micrographs were made using the bottom-mounted 4 × 4 K Eagle CCD-camera and converted to tif-format using the TIA software. Equivalent circle (ECD) and Feret minimum diameter (Feret min diameter) measurements were performed as described in [20] .

### Centrifugal liquid sedimentation (CLS)

CLS measurements were performed with a DC24000 system (CPS instruments Inc., Stuart, Florida, USA), equipped with a 405-nm wavelength laser detector, with polystyrene particles standard (nominal particle size = 10 μm) at a centrifugal speed of 2000 rpm. Speed was chosen in order to increase resolution of micron size aggregates. Sizes are expressed in terms of hydrodynamic diameter assuming all particles are spherical. Each measurement was done by injecting 1 ml of a 0.64 mg SAS/mL for SuperN or 1 ml of 10 mg/mL for PREC.

### Bright field (BF) microscopy

A drop of freshly prepared suspensions was placed on a slide and covered with a cover slip. The images were taken immediately using microscopy (BX61, Olympus, Belgium) in a BF mode and at a magnification of 40x. The diameter of 100 particles from the pictures was measured using an in-built arbitrary line measurement tool and the mean diameter was calculated.

### In vitro dosimetry

The Volume Centrifugation Method (VCM) was used to determine the effective density of SAS in cell exposure medium and the SAS concentration delivered to the cells over 24 h exposure was simulated using the Distorted Grid (DG) model as described in [30]. The results are presented as a function of nominal doses (presented as μg/mL).

### Inductively coupled plasma-mass spectrometry (ICP-MS)

Si was quantified directly in SAS SuperN and PREC suspensions by ICP-MS (Agilent 7500ce Octopole Reaction System, Santa Clara, USA) using a collision cell in helium mode, Sc as internal standard (Merck, Darmstadt, Germany) and a six-point calibration curve (Si standard, Merck). Measurements were carried out in 3 independent experiments on ^28^Si and ^45^Sc.

### Cell culture conditions

The human bronchial epithelial cell line (16HBE14o- or HBE) and the human monocytic cell line (THP-1) were kindly provided by Dr. Gruenert (University of California, San Francisco, USA). HBE cells were cultured in DMEM/F12 supplemented with 5% fetal bovine serum (FBS), 1% penicillin-streptomycin (P-S) (100 U/mL), 1% L-glutamine (2 mM) and 1% fungizone (2.5 g/mL) while RPMI 1640 supplemented with 10% FBS, 1% P-S (100 U/mL), 1% L-glutamine (2 mM) and 1% fungizone (2.5 g/mL) was used for THP-1. Caucasian colon adenocarcinoma cell line (Caco2) (P.Nr: 86010202) was purchased from Sigma- Aldrich (Belgium). DMEM/HG supplemented with 10% FBS, 1% P-S (100 U/mL), 1% L-glutamine (2 mM), 1% fungizone (2.5 g/mL) and 1% non-essential amino acids (NEAA) was used for Caco2 cells. All cell culture supplements were purchased from Invitrogen (Belgium) unless otherwise stated. Cells were cultured in T75 flasks (FALCON, USA) at 37 °C in a 100% humidified air containing 5% CO_2_. Fresh medium was changed every 2 or 3 days and cells were passaged every week. Cells from passage 4 to 10 were used for the experiments.

### In vitro exposure conditions

DE-AGGR and AGGR suspensions (2.56 mg/mL) were freshly prepared and diluted in BSA 0.05% to prepare different concentrations (20 μg/mL - 1280 μg/mL) and further diluted 10 times in serum-free exposure medium to achieve the final exposure concentrations (2–128 μg/mL). Regardless of mass concentrations in SuperN and PREC fractions, stocks were prepared and diluted exactly the same way as for AGGR and DE-AGGR suspensions. For toxicity testing, submerged cell cultures were used. For all experiments except TEER measurement, HBE, Caco2 and THP-1 cells were seeded at a density of 1.5 × 10^5^, 1.05 × 10^5^ cm^2^ (surface area of culture well) and 3 × 10^5^/mL respectively in 96 or 24 well plates (Greiner bio-one, Belgium). For the adherent cell-types (HBE and Caco2), the cell cultures were exposed as confluent cultures, to resemble as closely as possible NP exposure at the lung and intestinal epithelial barriers (in vivo and/or in humans). For non-adherent monocytic cell line (THP-1), the density 3 × 10^5^ cells/mL was selected based on the density of monocytes in 1 mL of human blood (3 × 10^5^ - 10 × 10^5^ monocytes/mL). After seeding and overnight incubation at 37 °C, cells were washed with HBSS (without Ca^2+^/Mg^2+^) once and exposed to different concentrations of SAS for 24 h. Cell cultures exposed to BSA 0.05% diluted ten times in serum-free exposure medium served as untreated control. After 24 h exposure, the cell cultures were washed twice with HBSS and the respective assays were performed.

### Cell metabolic activity

To determine cell metabolic activity, cell culture supernatants were removed after 24 h exposure and cells were incubated with 120 μL water soluble tetrazolium salts (WST-1) reagent (Roche, Belgium) diluted in medium without phenol red at the ratio of 1:10. After 1 to 2 h incubation at 37 °C, plates were centrifuged at 1600 *g* for 10 min, 100 μL was transferred to a new plate and the optical density (OD) was recorded using a micro-plate reader (Bio-Rad, USA) at 450 nm. After subtracting the blank OD values from the sample OD values, results were expressed as percentage of control (untreated) cells.

### Effect on cell viability

Effect on cell viability was assessed by cellular leakage of lactate dehydrogenase (LDH) using a kinetic assay [31]. At the end of exposure, supernatants were transferred to a new plate and cells were incubated with triton 0.2% (Sigma-Aldrich, Belgium). After 30 min, plates were centrifuged at 1600 *g* for 10 min. After transfer to a new plate, freshly prepared substrate solution (pyruvate + NADH) was added and the absorbance was measured by a spectrophotometer at 340 nm for 3 min with 15 s interval. Slope was calculated according to the standard curve. Cell viability was calculated as

$$ \left[\mathrm{slope}\ \mathrm{of}\ \mathrm{leakage}/\left(\mathrm{slope}\ \mathrm{of}\ \mathrm{lysate}+\mathrm{slope}\ \mathrm{of}\ \mathrm{leakage}\right)\ast 100\right]\ \mathrm{and}\ \mathrm{relative}\ \mathrm{viability}\ \mathrm{as}\ \left(\mathrm{sample}\ \mathrm{viability}/\mathrm{untreated}\ \mathrm{control}\ \mathrm{viability}\right)\ast 100 $$

### Total glutathione measurements

Total glutathione (GSH) is a cellular antioxidant, which is depleted when excessive reactive oxygen species (ROS) are produced. Therefore, GSH depletion was measured as an indicator of oxidative stress [32] using a GSH detection kit (Enzo life sciences, Belgium). After 24 h exposure, cell cultures were washed and harvested using trypsin 0.1% (Gibco, Belgium). Then, cells were resuspended in metaphosphoric acid 5% and homogenized using an ultra turrax t25 tissue homogenizer (Janke & kunkel, Germany). GSH was quantified according to manufacturer’s protocol and the protein content of cell cultures was assessed by the bicinchoninic acid (BCA) protein assay kit (Thermo Scientific Pierce, Belgium). GSH was normalized to the total protein content and the results were expressed as percentage of control (untreated) cells.

### Cytokine quantification

As indicators of pro-inflammatory responses, IL-8 and IL-6 levels were measured using enzyme-linked immunosorbent assay (ELISA) kits (Sigma-Aldrich, Belgium) in cell supernatants collected after 24 h exposure according to manufacturer’s protocol. Results were normalized to the total protein content and expressed as a ratio to control (untreated) cells. Cells treated with lipopolysaccharides 1 μg/mL were used as positive control (data not shown).

### Trans epithelial electrical resistance (TEER)

TEER was measured in epithelial (HBE and Caco2) monolayers as an estimation of epithelial barrier integrity. HBE and Caco2 cells were seeded at a density of 2.10^4^ cells per well in 24 well transwell inserts (0.4 μm pore size, polyester membrane, Corning, CLS3470 Sigma). TEER was monitored everyday using a Chopstick electrode and an epithelial voltohmmeter (EVOM) (World Precision Instruments, Sarasota, USA). After 7 d, cultures with TEER > 600 Ω.cm^2^ were exposed to different concentrations of SAS suspensions for 24 h and TEER was measured. Cultures exposed to sodium dodecyl sulfate (SDS) 200 μg/mL served as positive control for barrier integrity disruption (data not shown). Results were expressed as percentage of control (untreated) cells.

### Statistical analysis

Three independent experiments were performed in triplicate or duplicate and data were presented as mean ± standard deviation (SD). Using GraphPad prism 7 software (https://www.graphpad.com/), results were analysed with one-way ANOVA followed by a Dunnett’s multiple comparison test to determine the significance of differences compared to control. Two-way ANOVA followed by Bonferroni’s multiple comparison test was used to determine significance of differences between suspensions (see Table 4 for explanation).

## Supplementary information

**Additional file 1: Table S1.** Main parameters necessary to calculate the in vitro delivered doses for different SAS suspensions. Effective density of SAS in exposure media and, density and viscosity of exposure media prepared from DMEM/F12 and RPMI 1640 were calculated. All dosimetry simulations were performed for 24 h incubation at 37 °C and for 6 mm cell culture medium height. The hydrodynamic sizes are given in Table 2. **Figure S1.** Influence of SAS aggregation on cytotoxicity and biological responses. **Figure S2.** Influence of SAS aggregation on cytotoxicity and biological responses. **Figure S3.** Influence of SAS aggregation on cytotoxicity and biological responses.

**Additional file 2: Figure S4.** Effect of probe sonication energy on the size distribution of synthetic amorphous silica (SAS).

## Data Availability

The datasets used and/or analysed during the current study are available from the corresponding author on reasonable request.

## Abbreviations

ANOVA: Analysis of variance
BCA: Bicinchoninic acid assay
BSA: Bovine serum albumin
CCD: Charge coupled device
CCM: Cell culture medium
CLS: Centrifugal liquid sedimentation
DG: Distorted grid
DLS: Dynamic light scattering
ECD: Equivalent circle diameter
EDTA: Ethylenediaminetetraacetic acid
EFSA: European food safety authority
ELISA: Enzyme linked immunosorbent assay
FBS: Fetal bovine serum
Feret min: Minimum feret diameter
GSH: Total glutathione
HBSS: Hank’s balanced salt solution
ICP-MS: Inductively coupled plasma-mass spectrometry
IL: Interleukin
JRC: Joint research commission
LDH: Lactate dehydrogenase
NMs: Nanomaterials
NPs: Nanoparticles
OD: Optical density
OELs: Occupational exposure limits
PBS: Phosphate buffer saline
PDI: Polydispersity index
P-S: Penicillin-streptomycin
ROS: Reactive oxygen species
SAS: Synthetic amorphous silica
SD: Standard deviation
SDS: Sodium dodecyl sulfate
SEM: Standard error mean
TEER: Transepithelial electrical resistance
TEM: Transmission electron microscopy
VCM: Volume centrifugation method
WST-1: Water soluble tetrazolium salts 1
Z-average: Mean hydrodynamic diameter
ZS: Zeta sizer
