# Supplementary material for: Is aggregated synthetic amorphous silica toxicologically relevant?
Source: Part Fibre Toxicol. 2020 Jan 3;17:1. doi: 10.1186/s12989-019-0331-3 (PMC6942297; doi:10.1186/s12989-019-0331-3)
Supplement: Supplementary file 1 — Additional file 1: Table S1. Main parameters necessary to calculate the in vitro delivered doses for different SAS suspensions. Effective density of SAS in exposure media and, density and viscosity of exposure media prepared from DMEM/F12 and RPMI 1640 were calculated. All dosimetry simulations were performed for 24 h incubation at 37 °C and for 6 mm cell culture medium height. The hydrodynamic sizes are given in Table 2. Figure S1. Influence of SAS aggregation on cytotoxicity and biological responses. Figure S2. Influence of SAS aggregation on cytotoxicity and biological responses. Figure S3. Influence of SAS aggregation on cytotoxicity and biological responses. [file 12989_2019_331_MOESM1_ESM.docx]

**Additional file 1:**

**Table S1: Main parameters necessary to calculate the *in vitro* delivered doses for different SAS suspensions.**

|  | DMEM/F12 | | | RPMI 1640 | | |
| --- | --- | --- | --- | --- | --- | --- |
| Suspension | DE-AGGR | AGGR | SuperN | DE-AGGR | AGGR | SuperN |
| Effective density (g/cm^3^) | 1.2421 | 1.1415 | 1.0877 | 1.2644 | 1.1379 | 1.0984 |
| Medium density (g/cm^3^) | 1.0025 | 1.0025 | 1.0025 | 1.0050 | 1.0050 | 1.0050 |
| Medium viscosity (mPa S) | 0.98 | 0.98 | 0.98 | 0.98 | 0.98 | 0.98 |

Effective density of SAS in exposure media and, density and viscosity of exposure media prepared from DMEM/F12 and RPMI 1640 were calculated. All dosimetry simulations were performed for 24 h incubation at 37°C and for 6 mm cell culture medium height. The hydrodynamic sizes are given in table 2.

**Figure S1: Influence of SAS aggregation on cytotoxicity and biological responses.** Effect on cell metabolic activity (A, G, M), cell viability (B, H, N), total glutathione (C, I ,O), TEER (D, J), IL-8 (E, K, P) and IL-6 secretion (F, L, Q) measured in HBE (A-F), Caco2 (G-L) and THP-1 (M-Q) after 24 h exposure to DE-AGGR (red line) and AGGR (black line) suspensions. Data are expressed as means ± SD from three independent experiments performed in triplicates or duplicates. p < 0.05 (*), p < 0.01 (**) and p < 0.001 (***) represent significant differences compared to control (One-way ANOVA followed by Dunnett’s multiple comparison test). Two-way ANOVA was used to determine the significant difference between suspensions (significant p value indicated at the top left corner of the graph).

**Figure S2: Influence of SAS aggregation on cytotoxicity and biological responses.** Effect on cell metabolic activity (A, G, M), cell viability (B, H, N), total glutathione (C, I, O), TEER (D, J), IL-8 (E, K, P) and IL-6 secretion (F, L, Q) measured in HBE (A-F), Caco2 (G-L) and THP-1 (M-Q) after 24 h exposure to aggregated (AGGR, black line), supernatant (SuperN, blue line) and precipitated fraction (PREC, brown line) fractions. Data are expressed as means ± SD from three independent experiments performed in duplicates. p < 0.05 (*), p < 0.01 (**) and p < 0.001 (***) represent significant differences compared to control (One-way ANOVA followed by Dunnett’s multiple comparison test). The concentrations of SuperN and PREC suspensions were not calculated according to ICP-MS measurements (see results) and are presented identical to AGGR to better compare the effects of the individual fractions.

**Figure S3: Influence of SAS aggregation on cytotoxicity and biological responses.** Effect on cell metabolic activity (A, G, M), cell viability (B, H, N), total glutathione (C, I, O), TEER (D, J), IL-8 (E, K, P) and IL-6 secretion (F, L, Q) measured in HBE (A-F), Caco2 (G-L) and THP-1 (M-Q) after 24 h exposure to de-aggregated suspension (DE-AGGR, red line) and supernatant fraction (SuperN, blue line). Data are expressed as means ± SD from three independent experiments performed in duplicates. p < 0.05 (*), p < 0.01 (**) and p < 0.001 (***) represent significant differences compared to control (One-way ANOVA followed by Dunnett’s multiple comparison test). Two-way ANOVA was used to determine the significant differences between suspensions (significant p value indicated at the top left corner of the graph).
