# Supplementary material for: Is aggregated synthetic amorphous silica toxicologically relevant?
Source: Part Fibre Toxicol. 2020 Jan 3;17:1. doi: 10.1186/s12989-019-0331-3 (PMC6942297; doi:10.1186/s12989-019-0331-3)
Supplement: Supplementary file 2 — Additional file 2: Figure S4. Effect of probe sonication energy on the size distribution of synthetic amorphous silica (SAS). [file 12989_2019_331_MOESM2_ESM.docx]

**Additional file 2:**

**Figure S4: Effect of probe sonication energy on the size distribution of synthetic amorphous silica (SAS).** SAS were sonicated at different energies and analysed for feret minimum (feret min) using transmission electron microscopy (TEM) images. Energy 0 indicates that SAS were mechanically stirred using a vortex shaker instead of probe sonication.
